# Incidence and contributing factors of glucose intolerance in Saudi postpartum women: Sub-group analysis from RAHMA study

**DOI:** 10.1371/journal.pone.0210024

**Published:** 2019-01-07

**Authors:** Hayfaa Wahabi, Amel Fayed, Safaa M. S. Tunkar, Hanadi Bakhsh, Ali M. Al-Hazmi, Samia Esmaeil, Amna R. Siddiqui

**Affiliations:** 1 Chair of Evidence-Based Healthcare and Knowledge Translation, College of Medicine, King Saud University, Riyadh, Saudi Arabia; 2 Clinical Department, College of Medicine, Princess Nourah Bint Abdulrahman University, Riyadh, Saudi Arabia; 3 Department of Biostatistics, High Institute of Public Health, Alexandria University, Alexandria, Egypt; 4 Department of Family and Community Medicine, College of Medicine, King Saud University, Riyadh, Saudi Arabia; 5 Prince Sattam Bin Abdul Aziz Research Chair for Epidemiology and Public Health, College of Medicine, King Saud University, Riyadh, Saudi Arabia; 6 Department of Community Health Sciences, Aga Khan University, Karachi, Pakistan; Cincinnati Children's Hospital Medical Center, UNITED STATES

## Abstract

**Objectives:**

The objectives of this study were to determine incidence and risk factors of glucose intolerance one year after delivery in a sub-cohort of **R**iy**a**d**h M**other and B**a**by Cohort Study (RAHMA) study.

**Methods:**

This is a follow-up study of a sub-cohort from RAHMA study from King Khalid University Hospital (KKUH). All women from RAHMA database who completed one year since delivery at KKUH were contacted by phone to participate in the study. Previously collected data from RAHMA registry for each participant were linked to this study data. Clinical data measured for each participant included current weight and height to calculate the BMI and waist circumference. Blood tests done for each participant were fasting blood glucose (FPG) and HbA1c. Based on the blood tests results, participants were classified into three groups; diabetic, pre-diabetic and normal. The incidence of diabetes and prediabetes was calculated for the whole cohort. Clinical, biochemical, and sociodemographic predictors of glucose intolerance were compared between the three groups. Risk factors with P-value less than 0.05 were tested in multivariate regression model with bootstrapping to calculate the relative risk (RR) and its 95% Bias corrected Confidence Interval (C.I.)

**Results:**

From the sub-cohort, 407 women fulfilled the inclusion criteria and agreed to participate in the study. From the study participants; 250 (61.4%) women were normoglycemic, 142 (35%) women had prediabetes and 15 (3.6%) women were diabetic. Following multivariable regression analysis only history of gestational diabetes mellitus (GDM), (RR 1.74, 95% CI (1.06 to 2.84), P = 0.01), obesity (RR 1.69, 95% CI (1.01–3.11), P = 0.04) and diastolic blood pressure, (RR 1.04, 95% CI (1.01–1.09), P = 0.03) remained as predictors of postpartum glucose intolerance.

**Conclusion:**

The incidence of postpartum glucose intolerance (diabetes and prediabetes) is very high in Saudi women. Both GDM and obesity are strong predictors of glucose intolerance.

## Introduction

Diabetes is becoming a global health problem and is one of the main causes of premature morbidity and mortality [1].

Saudi Arabia is one of the top countries with the highest prevalence of diabetes [2]. More than 25% of Saudi adults are diabetic, with 40% of those are unaware of their condition [3]. In addition, 25% of the adult population are pre-diabetic [3]

The physiological changes of pregnancy create a state of carbohydrate intolerance mediated by the pregnancy specific hormones, which increase the peripheral resistance to insulin and call for more production of the hormone to maintain normal blood glucose levels during pregnancy [4].

One of the commonest glucose metabolism disorders of pregnancy is gestational diabetes (GDM), which is defined as diabetes diagnosed during pregnancy that is clearly not pre-gestational in nature [5].

It is proven that women with history of GDM are at increased risk of developing type 2 diabetes mellitus, metabolic syndrome and cardiovascular diseases later in life [6,7].

Most of the women with GDM revert to normal glycemic tolerance following delivery, however, they have seven-fold increased risk of developing type 2 diabetes compared to women who did not have GDM [8]. The reported rate of progression to type 2 diabetes varied considerably in different studies and is dependent on the period of follow up, the diagnostic criteria for GDM and ethnicity of the studied population [9]. Other factors such as family history of type 2 diabetes, severity of hyperglycemia during pregnancy, need of insulin treatment, and the anthropometric profile of the woman, are predicters of progression to type 2 diabetes in women with history of GDM [10–13]

Evidence suggests that lifestyle modification and pharmacological interventions can prevent or delay progression to type 2 diabetes in at risk population including women with history of GDM [14–16]. Hence, the identification of at risk women with history of GDM through postpartum screening programs, followed by appropriate interventions is expected to reduce the burden of type 2 diabetes, considering that one in four Saudi women develops GDM during pregnancy and is among the high-risk population for developing type 2 diabetes [17].

As per guidance of American diabetes association (ADA), different tests are equally effective for screening for prediabetes and diabetes including 75 oral glucose tolerance test (OGTT), fasting plasma glucose (FPG) or glycosylated haemoglobin A1C (HbA1c) [18].

There is paucity of studies on progression to type 2 diabetes among postpartum Saudi women. The objectives of this study were to determine incidence and risk factors of glucose intolerance one year after delivery in a sub-cohort of Riyadh Mother and Baby Cohort Study (RAHMA) study.

## Materials and methods

RAHMA is the first large multicentre, longitudinal cohort study which investigates pregnancy outcomes in Saudi Arabia. The main objectives of the study were to examine the influence of non-communicable diseases such as diabetes, hypertension, and obesity, on the mother and the baby. The participating hospitals were selected randomly after stratification based on the type of hospital (ministry of health, military, or teaching) and the number of beds which ensured that the participants in the study were representatives of all the spectra of pregnant women in Riyadh. The detailed methodology of the study has been previously reported [19]. In brief, participants completed a self-administered questionnaire providing information on family socioeconomic and lifestyle status and antenatal history. In addition, maternal and neonatal outcomes were reported. A link was established between maternal laboratory data and the study records [19].

This follow up study was conducted in a sub-cohort of participants of RAHMA from king Khalid University Hospital (KKUH), which is a tertiary referral hospital with 800 beds. It has all the essential departments including 20 operating theatres, an assisted reproduction unit and a cardiac centre. The hospital provides free medical care to Saudi nationals and the staff of King Saud University. The obstetrics department provides care for 3000–4000 deliveries per year [19]. The hospital does not provide routine postnatal screening for hyperglycemia for women with GDM.

### Definitions

- GDM was diagnosed based on one or more abnormal value of the 75g oral glucose tolerance test (OGTT) done between 24 and 34 gestation weeks using the following World Health Organization (WHO) cut-off values [20]:
  Fasting plasma glucose (FPG) 5.1–6.9 mmol/l (92–125 mg/dl).
  1-Hour plasma glucose ≥ 10.0 mmol/l (180 mg/dl).
  2-Hour plasma glucose 8.5–11.0 mmol/l (153–199 mg/dl).
- Diabetes and prediabetes were diagnosed using either fasting plasma glucose (if the participant came fasting) or glycosylated haemoglobin A1C (HbA1c) levels according to the following cut-off levels of the ADA [18]:
  Fasting Plasma Glucose (FPG) values:
    Normal < 5.6 mmol/l (100 mg/dl)
    Prediabetes 5.6–6.9mmol/l (100 mg/dl to 125 mg/dl)
    Diabetes ≥ 7.0 mmol/l (126 mg/dl)
  HbA1c values:
    Normal < than 5.7% (39 mmol/mol)
    Prediabetes 5.7% to 6.4% (39 to 46 mmol/mol)
    Diabetes≥ 6.5% (48 mmol/mol)
- Maternal pre-pregnancy body mass index (BMI) was calculated from maternal recall of weight prior to pregnancy and height measured during the first antenatal clinic. Current BMI was calculated using the current maternal weight and height. Participants were classified according to the WHO BMI definitions as follows: underweight (<18.5 kg/m2), normal weight (18.5–24.9 kg/m2), overweight (25.0–29.9 kg/m2) and obese (≥ 30 kg/m2) [21].
- A tape measure was used by one trained researcher to measure, waist circumference (WC) at the mid-point, half-way between the right iliac crest and the lower costal region [22].

### Study population

This is a follow-up study of subgroup of 3130 women from KKUH. All women who participated in RAHMA study from KKUH and met the inclusion criteria, were contacted one year after each woman’s delivery date to participate in the study. The inclusion criteria for this sub-cohort were the following:

1. Gestational age of 37–41 weeks at the time of delivery, calculated from the last menstrual period and/or early ultrasound scan, when there is a difference between menstrual date and ultrasound date the latter was taken as the correct date.
2. Singleton pregnancy

We excluded women with unknown glycaemic status, those with pre-GDM diagnosed before or during pregnancy, women who were pregnant at the time of this study and those who declined to participate in the study.

### Interventions

All women from RAHMA database who delivered one year before at KKUH and fulfilled the inclusion criteria, were contacted by phone to participate in this study. Women who agreed to participate were invited to attend RAHMA outpatient clinic (research clinic) fasting for at least eight hours before their appointment. The objectives of the study were explained again face-to-face to each participant who subsequently signed an informed consent form. Previously collected data from RAHMA registry for each participant were linked to this study data; including demographic profile, obstetric history such as parity, pre-pregnancy weight and type of treatment of GDM in the index pregnancy (diet or insulin). Clinical data measured in each participant included current weight and height to calculate the BMI in addition to waist circumference WC and systolic and diastolic blood pressure. Blood tests done for each participant were FPG and HbA1c levels.

### Outcomes measures

Based on the results of blood tests participants were classified into three groups; diabetic, pre-diabetic and normal. The incidence of diabetes and prediabetes was calculated in the whole cohort and the relative risk (RR) of the known risk factors was calculated.

### Statistical analysis

Statistical analysis was performed using SPSS version 21.0 (SPSS Inc., Chicago, IL, USA) for Windows. Descriptive statistics in terms of average ± Standard deviation for quantitative variables and frequency and percentage for qualitative variables were used. Clinical, biochemical, and sociodemographic characteristics of women who progressed to postpartum glucose intolerance were compared with women with normal glucose tolerance using Bivariate analysis. One-way Analysis of Variance (ANOVA) was used to compare quantitative variables among the three groups; normal glucose tolerance, impaired glucose tolerance and diabetics, and bootstrapping was conducted to improve precision of estimates. Chi-Square test was used to evaluate the association between categorical variables and Fisher’s Exact test was used when indicated.

Different logistic regression models were developed to test the adjusted RR of various risk factors using modified logistic regression model [23]. All models considered the impairment of glucose intolerance as a binary outcome and various covariates were adjusted for clinically significant confounders as maternal age and BMI. Bootstrapping of the regression models was performed with 1,000 samples and Bias Corrected and accelerated (BCa) 95% Confidence Intervals (C.I.) were reported. P-value of less than 0.05 was considered as statistically significant.

### Ethical approval

The study was conducted after the approval of King Saud University review board with approval letter number 15/0445/IRB. The study was conducted according to the principles expressed in the Declaration of Helsinki.

## Results

**Fig 1:**
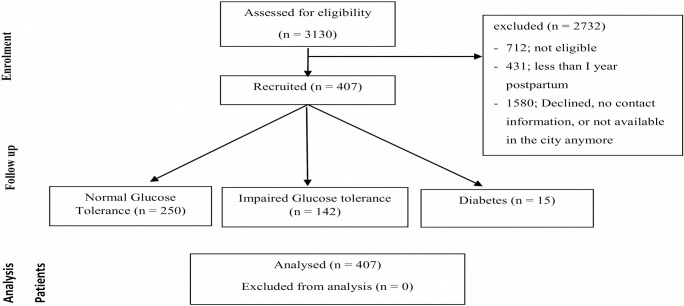
Recruitment and analysis of the cohort.

From a total of 3130 women, who delivered in KKUH and participated in RAHMA study, 712 women did not fulfil the inclusion criteria, 1067 women either declined to participate or had left the city, 513 women could not be contacted because they changed their contact information and 431 women were not yet one year postpartum when the study concluded leaving 407 women who fulfil the inclusion criteria and agreed to participate in the study (Fig 1).

Table 1 shows comparison of the main determinants of impaired glucose tolerance between women who participated in the study and those who did not. There was no significant difference between the two groups except for the prevalence of GDM.

**Table 1:** Comparison of characteristics of women who participated and those did not participate in the study.

	Participants N = 407	Non-participants N = 1580	p-value
**Age**	30.4±5.8	29.9±5.9	0.13
**parity**	3.17±2.4	3.19±2.2	0.88
**Pre-pregnancy BMI**	27.6± 5.9	27.9±5.7	0.34
**Term BMI**	31.7±6.2	31.2±5.6	0.25
**FBG**	4.6±2.8	4.7±1.8	0.50
**GDM**	142 (34.9)	285 (18.0)	<0.01

BMI, Body Mass Index; FBG, Fasting blood glucose; GDM, Gestational Diabetes

From the study participants; 250 (61.4%, BCa 95% C.I. = 57.2–66.1%) women were normoglycemic, 142 (35%, BCa 95% C.I. = 30.7–38.8%) women had prediabetes and 15 (3.6%, BCa 95% C.I. = 2.2–5.2%) women were diabetic.

Table 2 compares the characteristics of the three groups. From 133 women with history of GDM; 75 (56.4%) women developed either diabetes or prediabetes, while only 82 (30.1%) from 272 women without history of GDM developed prediabetes and none from this group developed diabetes (Table 2). There was no statistical difference between the three groups in parity (Table 2). However, normoglycemic women were significantly younger, had significantly lower mean BMI prior to the index pregnancy, compared to women with prediabetes and diabetes; (27.4 ±5.6, 28.3 ±5.5, 34.3 ±5.5 respectively, P<0.01). Similarly, the current mean BMI of normoglycemic women was significantly lower than that of the prediabetic and diabetic women (29.3 ± 6.2, 31.1 ± 6.6, 36.2 ± 4.8 respectively, P<0.01). Of the women who developed postpartum diabetes 14 (93.3%) had pre-pregnancy BMI of ≥ 30 kg/m^2^, while the corresponding proportion from the prediabetes group and the normoglycemic group was 66 (46.4%) and 108 (43.2%) respectively (Table 2). Measurements of WC and diastolic blood pressure, for women with prediabetes and diabetes, were significantly higher than those of normoglycemic women (Table 2). Significantly more women with prediabetes and diabetes had family history of diabetes, history of GDM in the index pregnancy, higher mean fating glucose level and 2 hours post glucose load. In addition, they more frequently needed insulin for control of GDM during pregnancy, compared to normoglycemic women (Table 2).

**Table 2:** Comparison of characteristics of women with normal glucose tolerance, impaired glucose tolerance and diabetes.

	Normal glucose tolerance (n = 250)	Impaired glucose tolerance(n = 142)	Diabetes (n = 15)	p-value
**Pre-pregnancy characteristics**				
**Age (years)**	31.6±5.5	32.6±6.2	35.6±5.8	0.02
95% BCa C.I.	30.2–31.8	31.1–33.6	31.2–37.5	0.04
**parity**				
primiparous	67 (26.9)	35 (24.8)	2 (13.3)	0.49
multiparous	183 (73.1)	107 (75.2)	13 (86.7)	
**Family history of type 2 diabetes**				
Yes	172 (68.7)	112 (78.7)	13 (86.7)	0.04
**Pre-pregnancy BMI**	27.4 ±5.6	28.3 ±5.5	34.3 ±5.5	<0.01
95% BCa C.I.	26.3–28.1	27.2–29.4	30.8–37.8	<0.01
BMI < 25	84 (33.6)	43 (30.4)	0 (0.0)	<0.01
BMI 25.1–29.9	58 (23.2)	33 (23.2)	1 (6.7)	
BMI ≥ 30	108 (43.2)	66 (46.4)	14 (93.3)	
**Pregnancy characteristics**				
**Pregnancy glucose level**				
Fasting	4.6 ± 0.69	4.8 ±0.64	5.5 ±0.77	<0.01
95% BCa C.I.	4.6–4.8	4.7–4.9	5.1–6.1	<0.01
I hour	7.6 ±1.9	7.9 ±2.2	8.1 ±2.2	0.28
95% BCa C.I.	7.22–7.87	7.37–8.61	8.91–10.92	0.16
2 hours	6.3 ± 2.1	6.6 ± 2.1	7.6 ±2.2	0.04
95% BCa C.I.	5.90–6.58	6.08–7.18	8.60–9.01	0.03
**GDM**				
Yes	58 (23.2)	60 (42.3)	15 (100.0)	<0.01
**Treatment of GDM**				
Diet	56 (96.6)	56 (93.3)	9 (60.0)	<0.01
insulin	2 (3.4)	4 (6.7)	6 (40.0)	
**Post-pregnancy anthropometric measurements**				
**Current BMI (kg/m**^**2**^**)**				
BMI (mean ±SD)	29.3 ± 6.2	31.1 ± 6.6	36.2 ± 4.8	0.01
**95% BCa C.I.**	28.0–29.9	29.7–32.2	33.9–39.7	<0.01
BMI< 25	69 (27.7)	26 (18.4)	0 (0.0)	<0.01
BMI 25.1–29.9	75 (30.1)	37 (26.2)	2 (13.3)	
BMI ≥ 30	105 (42.2)	78 (55.3)	13 (86.7)	
WC (mean ±SD) (cm)	82.4 ± 14.4	87.2 ± 13.0	89.2± 10.0	<0.01
95% BCa C.I.	76.26–79.85	79.76–85.67	87.22–94.07	<0.01
**Breast feeding (at 4 months)**	110 (44.0)	65 (45.7)	6 (40.0)	0.42
**Hormonal contraceptive use**	111 (44.4)	53 (37.1)	7(46.7)	0.34
**Systolic Blood Pressure**	112.9±13.5	113.4±17.5	115.2±14.6	0.80
95% BCa C.I.	110.61–114.99	108.53–116.92	112.85–121.90	0.83
**Diastolic blood pressure**	67.8±9.2	71.6±8.7	72.5±7.2	0.01
95% BCa C.I.	66.35–69.32	69.41–73.45	73.56–75.11	0.03

95% BCa C.I., 95% Bias Corrected and accelerated Confidence Interval; GDM, Gestational diabetes; BMI, Body Mass Index; WC, Waist circumference

All women who developed type 2 diabetes had history of GDM and none of the women who were normoglycemic during pregnancy developed type 2 diabetes. The RR of all factors for development of glucose intolerance is shown in Table 3. History of GDM in the index pregnancy almost doubled the risk of developing postpartum glucose intolerance; (RR 1.91, 95% confidence intervals (C.I.) (1.31 to 2.78), P<0.01). Similarly, women with BMI ≥ 30 kg/m^2^, had almost double the risk of developing postpartum glucose intolerance compared to women with lower BMI, (RR 1.79, 95% C.I. (1.17–2.97), P = 0.02) (Table 3). In addition higher diastolic blood pressure increased the risk of developing diabetes and IGT (RR 1.03, 95% C.I. (1.001–1.066), P = 0.04).

**Table 3:** Relative risk of developing glucose intolerance (prediabetes and type 2 diabetes) among the study cohort.

Risk Factor	RR (95% C.I.)	p- value	BCa RR(95% C.I.)	p-value
Age (years)	1.02(0.98–1.05)	0.41	1.01 (0.98–1.05)	0.43
**parity**				
primiparous	1			
multiparous	1.09 (0.69–1.69)	0.71	0.90 (0.55–1.49)	0.69
**Family history of DM**				
no	1			
yes	1.41 (0.90–2.20)	0.13	1.27(0.77–2.13)	0.31
**Pre-pregnancy BMI**				
Less than 25	1			
25.1–29.9	1.11 (0.63–1.94)	0.72	o.85(0.43–1.54)	0.59
30+	1.28 (0.81–2.03)	0.29	0.95 (0.55–1.69)	0.87
**Pregnancy fasting glucose level**	1.30 (0.99–1.69)	0.05	1.21 (0.91–1.69)	0.18
**GDM**				
No	1			
yes	1.91 (1.31–2.78)	<0.01	1.74 (1.06–2.84)	0.01
**Treatment of GDM**				
Diet	1			
insulin	1.37 (0.56–3.35)	0.48	1.49(0.53–3.85)	0.42
**Current BMI (kg/m**^**2**^**)**				
Less than 25	1			
25.1–29.9	1.31 (0.74–2.32)	0.34	1.22 (0.68–2.26)	0.49
≥ 30	1.79 (1.17–2.97)	0.02	1.69 (1.01–3.11)	0.04
**Diastolic Blood Pressure**	1.03 (1.001–1.066)	0.04	1.04 (1.01–1.09)	0.03

RR, Relative risk; BCa C.I., Bias Corrected and accelerated confidence interval; C.I., Confidence intervals; DM, Diabetes mellitus; GDM, Gestational Diabetes.

**Fig 2:**
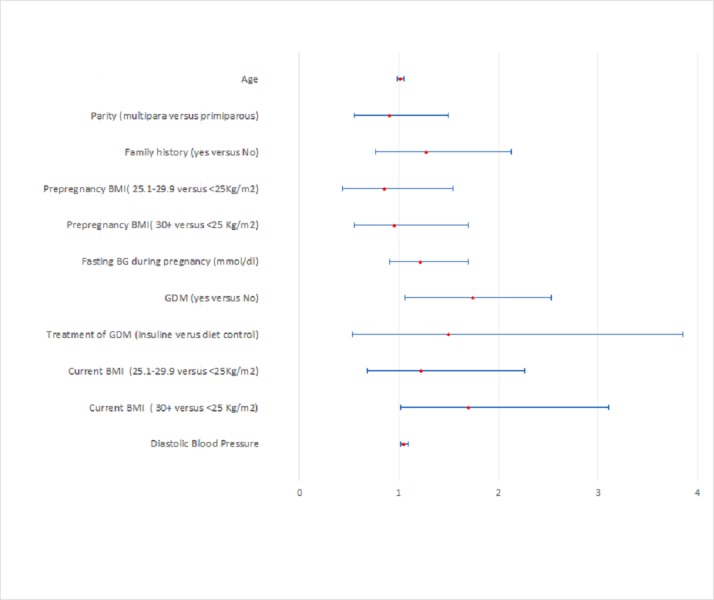
Forest plot of bias corrected relative risk for developing glucose intolerance.

However, in multivariable logistic regression analysis with bootstrapping, only history of GDM, (BCa-RR 1.74, 95% C.I. (1.06 to 2.84), P = 0.01), obesity (BCa-RR 1.69, 95% C.I. (1.01–3.11), P = 0.04) and diastolic blood pressure, (BCa-RR 1.04, 95% C.I. (1.01–1.09), P = 0.03) remained as predictors of postpartum glucose intolerance (Table 3 and Fig 2).

## Discussion

The incidence of prediabetes and diabetes, in this sub-group of RAHMA study, were 35% and 3.6% respectively. The results showed that women who developed glucose intolerance (diabetes and prediabetes), were significantly more obese, during pregnancy and in the postpartum period. Furthermore, a significantly higher proportion from this group had a history of GDM and a family history of type 2 diabetes. They had higher diastolic blood pressure and higher blood glucose levels during pregnancy and they needed insulin more frequently, for control of GDM, than normoglycemic group. The main predictors of developing glucose intolerance in this cohort were history of GDM, postpartum obesity and diastolic blood pressure.

This is, to our knowledge, the first study from Saudi Arabia to report on the incidence of glucose intolerance in postpartum women, one year after delivery. In a national survey of the general adult population in Saudi Arabia, the prevalence of prediabetes was estimated to be 25%, which is lower than the 35% rate found in this study. Moreover, the incidence (new cases) of diabetes in this study is higher than the incidence of 1.3% estimated previously for the same age group and gender by Al-Quwaidhi el al [24]. These differences in incidence and prevalence may be due to difference in the distribution of the risk factors between the general population and our cohort which included high proportion of women with GDM.

Recent reports from Italy on the prevalence of hyperglycaemia in women who had GDM showed rates similar to those found in our study (32% and 4% for prediabetes and diabetes respectively) [25]. However, reports from Iran showed lower rates than ours for prediabetes and higher rates for diabetes (17.5% prediabetes and 4.5% type 2 diabetes) [26]. These differences in rates can be explained by the variation in the criteria for diagnosing glucose intolerance, the duration of follow-up period, as both studies examined glycemic tolerance at 6–12 weeks rather than after one year as in our study, in addition to the ethnic differences between the studied population [8].

Similar to previous reports, this study showed that the risk factors for the development of impaired glucose tolerance were significantly more prevalent in women who developed diabetes and prediabetes compared to those who remained normoglycemic [8,9]. These include factors existing before the index pregnancy such as pre-pregnancy weight and family history of type 2 diabetes, factor which occurred during the index pregnancy such as the need to use insulin to control blood glucose level and high FBG; in addition, to postpartum factors such as obesity and blood pressure readings.

Nevertheless, when we controlled for confounders, our results showed that only GDM, postpartum obesity and diastolic blood pressure remained as significant risk factors for the development of glucose intolerance.

Long term follow-up of women with GDM showed a seven to ten-fold increase in risk of developing type 2 diabetes compared to women who were not diagnosed with GDM [6,27]. The results from a recently published study from Korea showed that history of GDM, as the sole risk factor, increased the risk of developing diabetes by 2.5-fold [28]. Moreover, women with the combination of history of GDM and obesity had the highest cumulative incidence of type 2 diabetes compared to women with combination of any other risk factors [28].

There is great variation in the global prevalence of GDM [29]. Recent reports from Saudi Arabia estimated that more than 25% of pregnant Saudi women develop GDM based on World Health Organization diagnostic criteria [17,20]. Such high rate of GDM will continue to contribute to the epidemic of diabetes and its complications in the country [30] unless a robust screening program and appropriate interventions are established for this at-risk population [14,15].

Obesity was another predictor of progression to prediabetes and diabetes in this study. Similar results were reported by previously published studies [31,32]. Obesity imposes risk equal to that of GDM for developing hyperglycemia in postpartum women [28]. In addition, women with GDM who are obese, have 1.5-fold increase in the risk of developing diabetes in postpartum period compared to those who have normal BMI [32]. Recent reports from Saudi Arabia showed that more than 68% of women in the reproductive age group are either overweight or obese [19], and that the prevalence of GDM and Pre-GDM increases with the increase in BMI [17].

Another risk factor for persistent hyperglycemia in the postpartum period is diastolic blood pressure. Although blood pressure levels for all groups in this study were blew those of hypertension [33], but higher diabolic blood pressure was a predictor of development of hyperglycemia. Similar results were reported by Vambergue et al who speculated that most of the women who develop hyperglycemia following GDM will develop hypertension later in life [34]

Based on a recently published systematic review, lifestyle interventions, including healthy eating and physical activity, are effective for the treatment of women with GDM [35]. Women who received lifestyle interventions were more likely to achieve the target weight in the postnatal period and their neonates were less likely to be large for gestational age [35]. Furthermore, lifestyle interventions following delivery were effective in the prevention of progression to type 2 diabetes [36].

### Strength and limitations of the study

This study is the first to quantify the problem of glucose intolerance following GDM in a Saudi population. It provides valuable information for policy makers and researchers about the risk factors for progression to type 2 diabetes in Saudi women. However, the study population may not be representative of the whole population especially that there were significantly more women with GDM in the study group compared to the women who did not participate in the study (Table 1). This may be due to the fact that women who had pregnancies complicated with GDM were concerned about their health and were more willing to participate in the study than those who had normal pregnancy. Another limitation of this study is the relatively small number of participants compared to the total number of women who participated in RAHM from KKUH. This is due to the poor return of participants from the first cohort. Poor postpartum response to recall for follow-up is faced by other researchers in the Middle East [37]. Many factors contribute to this phenomenon including inadequate referral system, lack of national health information system, and lack of national registries in addition to the lack of the culture of longitudinal research in many of the Middle Eastern communities.

### Implication to practice

- Establishing a national program for screening adults, especially for women with history of GDM, for diabetes and prediabetes is a prudent step towards controlling the epidemic of diabetes in the country. Such programs were proven to be cost-effective given the saving in averting the cost of morbidity and premature death from complications of diabetes [38].
- Implementation of evidence based strategy for the treatment of GDM that includes lifestyle modification is an essential step in the control of postpartum progression to type 2 diabetes
- It is important to increase the awareness of Saudi women in the reproductive age group about the risks related to obesity and GDM and the importance of postnatal screening

### Implication to research

Research should be directed towards effective interventions to reduce the burden of obesity in Saudi pregnant and postpartum women.

## Conclusion

The incidence of postpartum glucose intolerance (diabetes and prediabetes) is very high in Saudi women. Both GDM and obesity are strong predictors of glucose intolerance.
