# Correction: Incidence and contributing factors of glucose intolerance in Saudi postpartum women: Sub-group analysis from RAHMA study

**DOI:** 10.1371/journal.pone.0213466

**Published:** 2019-02-28

**Authors:** Hayfaa Wahabi, Amel Fayed, Safaa M. S. Tunkar, Hanadi Bakhsh, Ali M. Al-Hazmi, Samia Esmaeil, Amna R. Siddiqui

In the Author Contributions, Safaa M. S. Tunkar should be listed as one of the authors who contributed to the conceptualization, investigation, and methodology of the study.
